# Effects of the Herbicide Glyphosate on Honey Bee Sensory and Cognitive Abilities: Individual Impairments with Implications for the Hive

**DOI:** 10.3390/insects10100354

**Published:** 2019-10-18

**Authors:** Walter M. Farina, M. Sol Balbuena, Lucila T. Herbert, Carolina Mengoni Goñalons, Diego E. Vázquez

**Affiliations:** 1 Laboratorio de Insectos Sociales, Departamento de Biodiversidad y Biología Experimental, Facultad de Ciencias Exactas y Naturales, Universidad de Buenos Aires, Buenos Aires 1428, Argentina; msbalbuena@bg.fcen.uba.ar (M.S.B.); lucilaherbert@gmail.com (L.T.H.); caromengoni@bg.fcen.uba.ar (C.M.G.); diegovazquez@bg.fcen.uba.ar (D.E.V.); 2 Biología Molecular y Neurociencias (IFIBYNE), Instituto de Fisiología, CONICET-Universidad de Buenos Aires, Buenos Aires 1428, Argentina

**Keywords:** *Apis mellifera*, agrochemical, glyphosate, cognitive abilities, behavior, brood development

## Abstract

The honeybee *Apis mellifera* is an important pollinator in both undisturbed and agricultural ecosystems. Its great versatility as an experimental model makes it an excellent proxy to evaluate the environmental impact of agrochemicals using current methodologies and procedures in environmental toxicology. The increase in agrochemical use, including those that do not target insects directly, can have deleterious effects if carried out indiscriminately. This seems to be the case of the herbicide glyphosate (GLY), the most widely used agrochemical worldwide. Its presence in honey has been reported in samples obtained from different environments. Hence, to understand its current and potential risks for this pollinator it has become essential to not only study the effects on honeybee colonies located in agricultural settings, but also its effects under laboratory conditions. Subtle deleterious effects can be detected using experimental approaches. GLY negatively affects associative learning processes of foragers, cognitive and sensory abilities of young hive bees and promotes delays in brood development. An integrated approach that considers behavior, physiology, and development allows not only to determine the effects of this agrochemical on this eusocial insect from an experimental perspective, but also to infer putative effects in disturbed environments where it is omnipresent.

## 1. Background: The Presence of the Herbicide Glyphosate in the Surroundings of Honey Bee Hives

Estimating the environmental impacts that take place in disturbed ecosystems such as agricultural settings requires a multidisciplinary perspective that integrates different approaches to achieve a real understanding of anthropic action [1,2,3]. The current technology package that arose from the Green Revolution maximizes yield and benefits farmers in less time. However, it also affects natural and disturbed ecosystems as a whole, and its impact is growing [3,4,5]. The presence of agrochemicals has increased in the last decades in terms of cropland surface and amount of product used per surface area [6]. Thus, even those agrochemicals that do not directly target certain organisms can have pernicious effects on them if used indiscriminately.

Insect pollinators are one of the non-target organisms impacted by the use of agrochemicals [5,7]. They have gained growing attention due to evidence that shows the negative impact that intensive agricultural practices have on them and on pollination itself [8]. Landscapes exposed to monocropping and industrial agriculture positively correlate with an indiscriminate use of pesticides, which has clear negative effects on insect pollinator abundance and diversity [8]. Bees are essential pollinators of plants with flowers [8,9] and *Apis* bees are one of the most abundant pollen vectors used by beekeepers in agricultural settings [10]. Honey bees *Apis mellifera* are present in very diverse ecosystems and their great versatility as experimental models under controlled laboratory conditions make *A. mellifera* an excellent species to evaluate the effects of environmental changes that might currently be undetected with many of the endpoints used for product registration in environmental toxicology [11]. Due to its social nature, the impact of the ecotoxicological results using the honeybee has implications that exceed a mere individual level analysis [12].

Glyphosate (GLY) is the most widely used agrochemical in the world [6,13] and has become a chemical model for the evaluation of potential deleterious effects of herbicides on non-target organisms. Although GLY has been considered nontoxic for honeybees on the basis of acute contact and oral toxicity tests (>100 µg acid equivalent, henceforth: a.e., per bee) [14], this herbicide indirectly affects pollinators by reducing resource availability as it harms flora present in the environment [7]. More alarming than the indirect effects it has are its direct ones, e.g., changes in pollinator gut microbiota and greater susceptibility to pathogens and malnutrition [15,16,17].

Insect pollinators can be exposed to GLY due to its presence in the surroundings of their nest. Several routes of exposure to agrochemicals—including this herbicide—can occur by contact with spray drift or contaminated dust, and by ingestion of residues in vegetation and water bodies [8,18,19]. GLY has a moderate persistence in the environment because its degradation depends on microbial activity in contaminated substrates. Abiotic degradation such as photolysis and hydrolysis slightly contribute to its dissipation [13]. GLY is generally less persistent in water than in soil, with a half-life in water which varies from a few days to 91 days [20]. Consequently, this herbicide could affect non-target organisms several days post-application.

Environmental chemistry studies show concentrations measured in water bodies close to agricultural settings ranged from a few micrograms to 1.7 mg·L^−1^ [13,19,21,22]. For the worst case scenario in small water bodies (ponds or puddles), a median expected environmental concentration of 3.49 mg·L^−1^ was calculated [23] and some residue measures of around 2.8 mg·L^−1^ have been reported [21,22]. In addition, herbicide traces are dissolved in plant phloem as a consequence of its systemic mode of action [20,24]. Death of susceptible vegetation may take from 4 to 20 days to occur [20]. Meanwhile, tolerant crops or resistant weeds could contain more GLY molecules inside [13,24]. Hence, food sources for pollinators from this vegetation, such as nectar and pollen, can be contaminated. GLY concentrations measured a few days after its application ranged from 2.78 to 31.3 mg·kg^−1^ in nectar and from 87.2 to 629 mg·kg^−1^ in pollen, with declination over time [25]. However, these values vary depending on plant species and environmental conditions.

Inside *Apis* colonies, after bees collect food from contaminated sources, this herbicide could finally concentrate because nectar is evaporated and condensed by food processor bees to make honey and bee bread [12]. GLY concentration could be modified during the fermentation of pollen (bee bread), but honey has a low bacterial charge due to the addition of enzymes by food processors and to other chemical properties [26]. In surveys of human food, GLY was present in samples from packaged honey in markets [27,28] and was also found in honey samples taken directly from beehives located in apiaries [29]. These three studies show average concentrations of 66, 77, and 118 µg·kg^−1^ of GLY in honey, respectively. Meanwhile, colonies restricted to forage only in flowers sprayed with the herbicide under experimental semi-field conditions showed accumulation of GLY in honey up to 1.3 mg kg^−1^ [25]. This herbicide has been detected in as many as 70% of packaged honey samples from countries where genetically modifies crops (GMO) are allowed [27]. However, GLY has even been detected in 46% of samples labelled as organic honey with an average concentration of 50 ng·kg^−1^ [27]. This indicates the accidental exposure to which bees are subject, presumably due to their wide maximum foraging range from 3.7 to 6 km, depending on the environment [12].

Although other pesticides including herbicides have been detected in honeybee brood food, such as royal jelly and wax combs, there is little information available that shows the presence of GLY in these beehive products [30]. It is relatively difficult to detect GLY and AMPA (its degradation metabolite) using conventional methods because their physical and chemical properties [31]. For this reason, it is important to continue developing reliable analytical methods to study the fate and levels of GLY in beehives samples due to the complexity of substrates. An increase in concentrations of GLY and other agrochemicals can be expected due to the agricultural intensification and use of GMO technology in many countries, which has had an exponential growth in the last decades [6,32]. Furthermore, GLY is also used on non-GMO crops and in non-agricultural environments (e.g., farmyard areas, car parks and verges, industrial complexes, and railway tracks, among others), summarizing around 50% of the total GLY consumed worldwide [6].

To determine the effects of GLY and other agrochemicals on pollinators, it is pertinent to investigate not only the impacts on bees in agricultural environments, but also to test bees’ responses under laboratory conditions. Social bee colonies present in agricultural environments experience real-world conditions, but laboratory investigations allow to detect subtle impacts and infer potential risks that might otherwise be masked in more complex contexts. Recent experimental evidence describes detrimental sub-lethal effects of GLY on different aspects of honey bee behavior, physiology, and development due to its ingestion and part of them are reviewed in this manuscript [33,34,35,36]. These effects are manifested at different moments of this pollinator’s lifespan, which implies an effect on the different tasks that a honey bee worker performs throughout her adult life within the colony.

Essentially, the cyclical foraging activity of *Apis mellifera*, the stability of GLY in stored honey and the great capacity of honey bees to distribute food rapidly among hive mates, make it necessary to evaluate how the exposure to this xenobiotic affects individuals with different degrees of development. This review focuses on how GLY affects foraging behavior of honey bee workers and its implications at different levels within the colony [33,34,35,36].

## 2. Searching for Food in Disturbed Environments: Changes in Cognitive Abilities Represented by Elemental and Non-elemental Olfactory Learning

Learning and remembering are essential for honey bees [37]. Foraging behavior alone relies heavily on these processes. Identifying a food source, e.g., a flower, and returning to it after successive flights to and from the hive depends on associating a resource, e.g., nectar or pollen, to certain floral cues such as odor, colour, quality or profitability [38]. Remembering this association enables honey bees to return to feed from a resource even if it is found within different floral patches or throughout different flowering events. In turn, this increases a colony’s possibilities of foraging in a precise and efficient way [12,37,39,40]. In particular, honeybees learn olfactory cues while visiting flowers that offer nectar as a reward [41,42,43].

Foraging in an agricultural context where GLY is present, honey bees could possibly come across GLY on both crop flowers and surrounding flowers (native or exotic) [24,25], which means that they could be exposed to the herbicide simultaneously with learning events. Moreover, forager bees show a preference for food sources with low concentrations of GLY [44]. As honeybees do not avoid foraging from GLY contaminated sources, exposure is repeated each time they revisit [33]. Honeybees establish predictive relationships between events that take place concurrently in their environment, and learn which stimuli are relevant through associative learning [41,42,43]; what could happen if GLY were present during this process?

**Figure 1:**
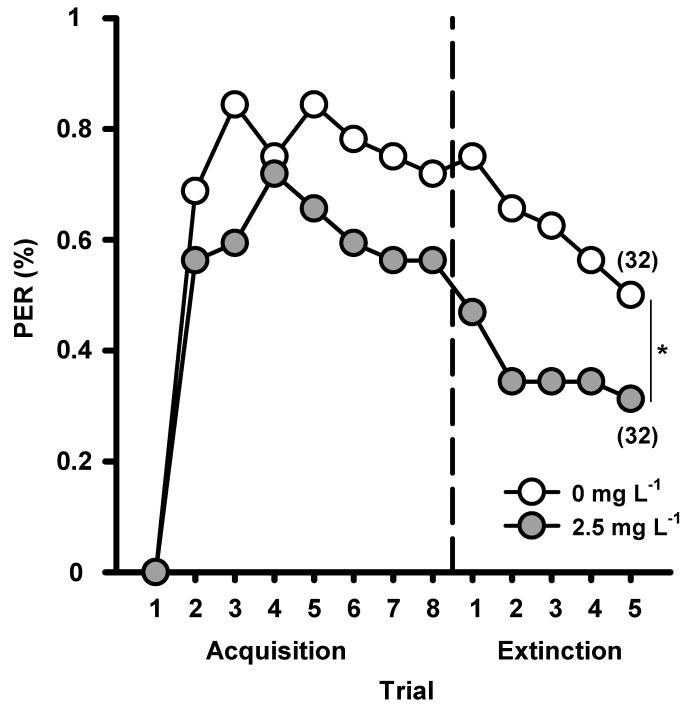
Effect of an acute exposure to GLY on forager honey bee elemental olfactory learning. Percentage of bees captured at the hive entrance that responded to the conditioned stimulus (CS) by extending their proboscis (% PER) in an absolute classical conditioning procedure was quantified over the course of eight acquisition and five extinction trials. During the acquisition phase, bees were offered either sucrose solution alone (control; white) or sucrose solution with 2.5 mg L^−1^ GLY (grey), as unconditioned stimulus. The extinction phase, during which the CS was not rewarded, started after the acquisition phase. Numbers in brackets indicate the number of bees in each treatment. Mann–Whitney: * *p* < 0.05. Reproduced with permission from Herbert and co-workers, Journal of Experimental Biology; published by The Company of Biologists, 2014.

Elemental associative learning occurs when a bee learns a unique and specific connection between a neutral stimulus such as a certain floral odor, and a reward such as nectar, and strengthens this association throughout different foraging events [41,42,43]. The effect of GLY on this type of associative learning was evaluated on foragers captured at a hive entrance by using a classical conditioning protocol of the proboscis extension response (PER) [33]. Honeybees were presented with an odor as neutral stimulus and, as unconditioned stimulus, sucrose solution with 2.5 mg·L^−1^ GLY or without (control). The ability to establish an elemental association between an odor and the sucrose reward was impaired by an acute exposure to GLY and this response was consistent throughout the course of repeated events (Figure 1, acquisition trials). Furthermore, honeybees that learned concomitantly with GLY showed a faster extinction process of these associations (Figure 1, extinction trials), which implies that GLY also diminishes short-term memory retention.

**Figure 2:**
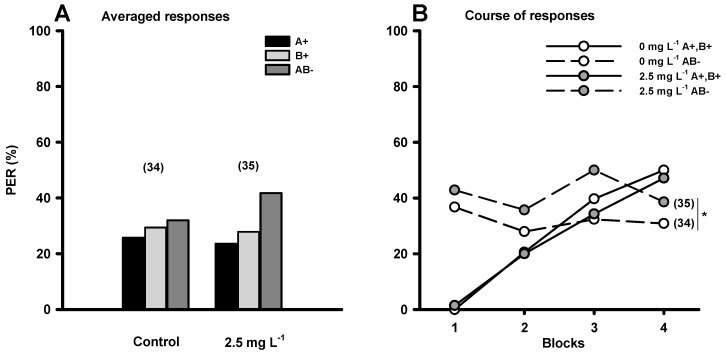
Effect of an acute exposure to GLY on forager honey bee non-elemental olfactory learning. Percentage of bees captured at the hive entrance that responded to the rewarded conditioned stimuli (CS+; A+, B+) and the unrewarded conditioned stimulus (CS−; AB−) by extending their proboscis (% PER) in a negative patterning olfactory conditioning procedure. The unconditioned stimulus consisted of either sucrose solution alone (control) or sucrose solution with 2.5 mg L^−1^ GLY. (**A**) Average of % PER for each treatment across all trials of A+, B+ and AB− for both groups. (**B**) % PER to the rewarded stimulus (A+, B+; solid lines) and to the unrewarded stimulus (AB−; dashed lines) for both treatments. The number of bees evaluated in each treatment is shown in brackets above each group of bars (**A**) and beside each curve (**B**). * *p* < 0.05 (two-way ANOVA). Reproduced with permission from Herbert and co-workers, Journal of Experimental Biology; published by The Company of Biologists, 2014.

In the natural world, floral cues rarely appear isolated. Normally, flowers combine several odors and this combination represents a new sensory cue for a honeybee [45,46,47]. Two floral species that have different nectar productivity may present a same chemical odor, but the floral scent of each species may be comprised by a different combination of chemical odors [48]. An odor can thus be present in a floral species with high nectar productivity and also in another species with low productivity. Honeybees can detect this difference and associate the specific combination of odors with the floral species with higher productivity. In these cases, there is no unique connection between an odor and the reward; odors are present in the honey bee’s perceptual world both associated and not associated to the reward. This non-elemental associative learning requires a more complex cognitive process, which could be more susceptible to GLY. To evaluate this, Herbert and co-workers (2014) used a negative patterning conditioning protocol [45,46,47]. Two individual odors were presented as conditioned stimuli concurrently with sucrose solution either alone (control) or with 2.5 mg·L^−1^ of GLY (rewarded odors: A+ and B+). The compound formed by the combination of the individual odors was also presented as conditioned stimulus, though unrewarded (non rewarded odor: AB−). Honeybees from both groups were able to successfully learn to associate the individual odors with reward (Figure 2A). They were also initially able to discriminate the reinforced elements (Figure 2B) from the non-reinforced element (Figure 2B), regardless of exposure to GLY. However, overall differentiation decayed in honeybees exposed to GLY along successive trials (Figure 2B) impairing the ability of honeybees to discriminate AB as a different entity from the simple sum of A and B.

Overall, forager honeybees acutely exposed to GLY need more learning events to establish elemental associations between an odor and a reward and present difficulties in discriminating a rewarded odor from an unrewarded mixture that contains the same odor in non-elemental associations. In turn, memories formed are weaker and can be extinguished more rapidly. GLY could be acting directly on chemo-sensory stimuli perception or on associations established between stimuli; the mechanism remains unknown. Continuous foraging of nectar with GLY traces present in agricultural environments has detrimental sub-lethal effects on their learning abilities which, if exposure were to occur in a similar manner as tested in the assays, could impact resource gathering, the coordination of collective activities and overall long-term colony survival.

## 3. Homeward Flights after Feeding from Resources Containing GLY Traces

During exploratory orientation flights, honeybees become familiar with the sun compass, measurement of distances and landmarks [49,50]. Further information about the landscape is added during flights between the hive and the feeding sites. Integration of the multiple sources of spatial information leads to a reference memory that allows bees to perform shortcuts between important locations. As a result, honey bees are able to refer to a common frame of spatial reference that allows them to return to the hive even when departing from an unfamiliar location by taking novel shortcuts [51]. This is a highly complex process that requires bees to memorize information coming from diverse sensory modalities.

**Figure 3:**
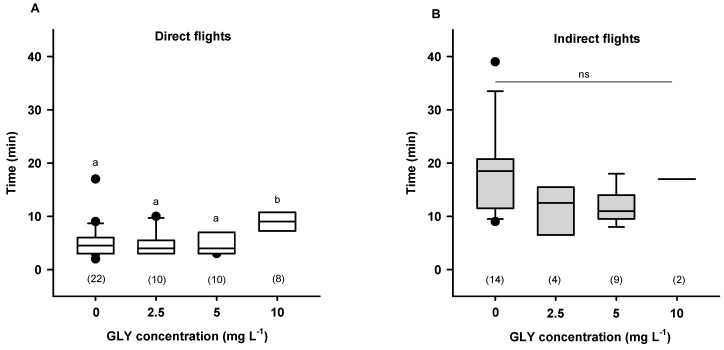
Effect of an acute exposure to GLY on forager homeward flight time after first release. Flying time (min) of bees from the release site to the hive after feeding sucrose solution with and without GLY (0 mg L^−1^: control, 2.5 mg L^−1^, 5 mg L^−1^, 10 mg L^−1^). (**A**) Direct flights. (**B**) Indirect flights. Different letters represent statistically significant differences (*p* < 0.05, Kruskal–Wallis test). ns: no significant differences (*p* > 0.05, Kruskal–Wallis test). Numbers in brackets indicate the number of bees tested for each treatment. Reproduced with permission from Balbuena and co-workers, Journal of Experimental Biology; published by The Company of Biologists, 2015.

With the aim of answering if the presence of GLY promotes a difficulty to integrate complex environmental information, which bees need for navigation, Balbuena and co-workers [34] performed a catch-and-release experiment. Honey bees were trained to an artificial feeder located 400 m from the hive. Once they landed on the feeder, bees were caught and fed sucrose solution containing different GLY concentrations (0 mg·L^−1^ for control and between 2.5 and 10 mg L^−1^ for exposed bees) and moved in individual black boxes from the feeding site to an unfamiliar release site. After one hour, a period that ensured uptake of the sucrose solution with or without the herbicide, bees were released and their homeward flights were tracked using harmonic radar. Both control and GLY fed bees performed two types of homeward flights: direct (straight flight from the release site to the hive) or indirect ones (flight with several loops before to arrive to the hive). Bees that ingested sucrose solution containing 10 mg·L^−1^ of the herbicide spent more time performing direct flights from the unfamiliar release site to the hive than control bees or those exposed to 2.5 or 5 mg·L^−1^ GLY (Figure 3, left panel). Indirect flight time was similar among control bees and those fed with any of the herbicide concentrations offered (Figure 3, right panel).

**Figure 4:**
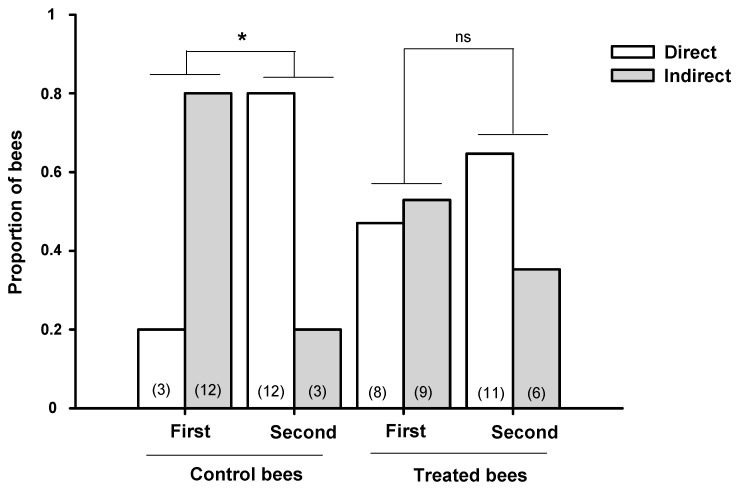
Effect of an acute exposure to GLY on forager transitions from direct to indirect homeward flights after two releases. Proportion of bees performing direct and indirect flights after the first and the second release after feeding sucrose solution with or without GLY (0 mg L^−1^ for control and 2.5 to 10 mg L^−1^ for treated bees). White bars: direct flights; grey bars: indirect flights. * *p* < 0.05 (Fisher’s exact test). ns: no significant differences (*p* > 0.05, (Fisher’s exact test). Numbers in brackets indicate the number of bees tested for each treatment. Reproduced with permission from Balbuena and co-workers, Journal of Experimental Biology; published by The Company of Biologists, 2015.

In addition, Balbuena and co-workers [34] studied the homeward flights after a second release from the same release site. Thus, the unfamiliar release site became a familiar one. Since honeybees improve homing flights across sequential releases from the same location [49], it was expected that bees familiarized with environment landmarks would perform more direct flights back to the hive. The proportion of bees performing direct and indirect flights after one or two releases from the same release site was compared using the same catch-and-release methodology. In this case, a second release meant that bees were fed with sucrose solution, with or without GLY, a second time. Control bees perform more direct flights after the second release (Figure 4, control bees). However, the proportion of GLY fed bees performing direct and indirect flights did not change after a second release (Figure 4, treated bees).

These results show that GLY exposure affects the homeward flights of foragers. First, for high herbicide concentrations, the time spent to return to the hive after one release was higher. Second, bees that ingested GLY kept performing a high proportion of indirect flights after a second release. Thus, the exposure to the herbicide could impair learning, impact memory retrieval, or affect manoeuvres, all of which are necessary to successful foraging. The presence of disoriented foragers could affect the availability of active foraging bees with the consequent reduction in the honeybee population during the period in which the resources are available.

To summarize, GLY concentrations tested around the values of worst-case scenario, which were measured in water and nectar, showed sub-lethal effects and were not rejected by the forager bees, even after repeated exposure [33,34,44]. This indicates that, at least in the short term, foragers can collect contaminated food and carry it to the hive. Accordingly, Thompson and co-workers [25] measured increasing GLY concentration in honey stored in hives where colonies were restricted to forage only in vegetation sprayed with the herbicide. Forager bees visited these flowers during a week with declination in GLY concentration over time in the food sources. Although four days after the application there were no detectable residues in honey (<0.3 mg·kg^−1^), seven days later they reached an average concentration of 0.99 ± 0.15 mg·Kg^−1^ [25]. This means that the contaminated resources are quickly and massively accumulated within the nest causing a spatial concentration with increasing exposure of bees inside the nest.

## 4. How Cognitive Abilities of Young Hive Bees Are Affected by GLY Traces

As honeybees that collect nectar with GLY traces do not interrupt their foraging activity, a constant inflow of GLY is very likely in exposed colonies. Thus, once a forager returns to the hive, she can contaminate her nest mates through body contact, by sharing food directly or through the collected resources that are eventually stored in cells [52,53]. In fact, as mentioned before, reports confirm the presence of GLY traces in honey samples [25,27,28,29]. This way, in-hive workers and brood come into contact with this herbicide within the colony.

As colony survival depends on collective tasks, it is important to take into account the exposure of bees that remain inside the hive. These pre-foraging workers perform in-hive tasks that guarantee colony care and maintenance [12]. They feed on incoming resources, as well as those stored in combs. During this period, and until they perform their first flights, bees do not defecate, and therefore hold fluid and feces [54] with potential toxic metabolites. They also present changing physiology and anatomy [55,56] and exhibit high physiological and behavioral plasticity [57]. The high connectivity among colony mates within a beehive facilitates information flow. Young honeybees receive cues from the outside, especially those perceived through taste and smell [58]. The experiences these young bees undergo inside the hive can affect tasks they perform in the future, both inside and outside the nest [59].

**Figure 5:**
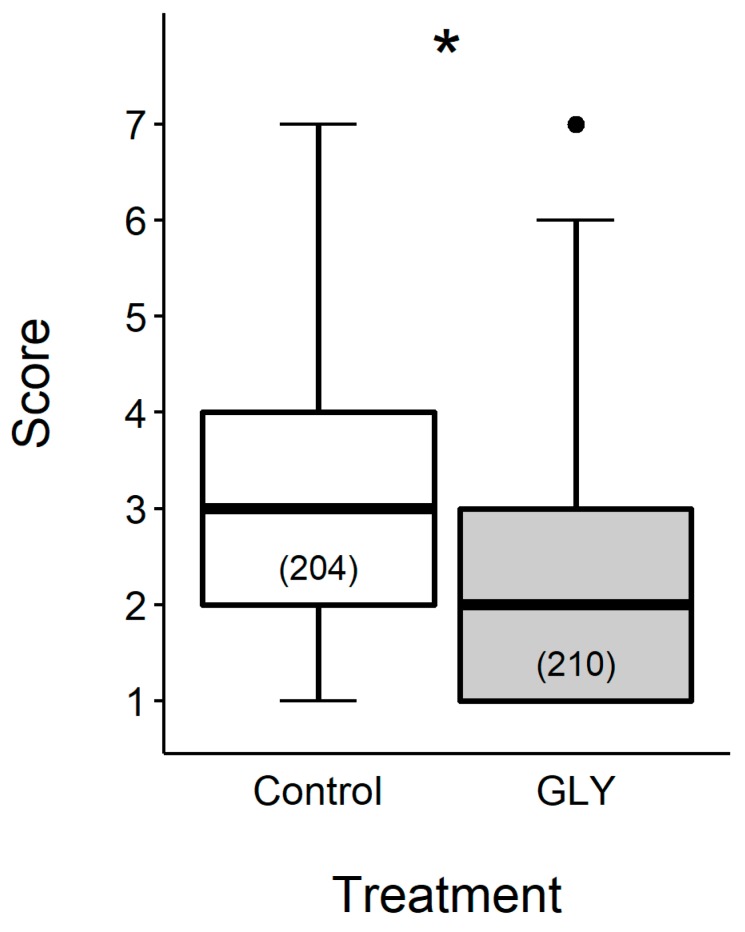
Effect of chronic exposure to GLY on young honey bee sucrose responsiveness. Gustatory response score of five, nine and fourteen day old bees reared in cages that were offered sucrose solution alone (control) or with GLY (2.5 mg L^−1^). Thick line, box and whiskers represent median, inter-quartile range and data range excluding extreme data (points), respectively. Numbers inside boxes indicate sample size. * indicates an effect of GLY (therefore, differences between its two levels), given that this factor is included in the minimal adequate model (MAM). Minimal adequate model (GLMM with binomial structure): Score ∼GLY + cage. Black circle represents outleir.

Contact chemoperception—or taste—allows insects to choose their food carefully. When collecting or processing nectar, pollen, water, and plant resins, honey bees come in contact with numerous compounds which are detected by gustatory organs. Stimulation of these chemosensory organs with sucrose elicits the reflex behavioral response PER [60]. Honeybees’ sucrose responsiveness can be tested by offering them solutions of increasing sucrose concentration and evaluating the overall score, defined as the sum of PER throughout the procedure [61]. Chronic exposure to GLY has an influence on responsiveness [33,35] and its effect does not depend on honeybee age. Honey bees fed with sucrose solution plus GLY had lower scores than control bees, which is interpreted as a rise in their sucrose response thresholds (Figure 5). Older pre-foraging workers typically act as nectar receivers, and the chance of accepting the resource gathered by foragers depends on their individual threshold. This decision affects food distribution within the hive [62,63,64]. Constant inflow and storage of GLY contaminated nectar, which in-hive workers feed on, could slow down general supply of energy sources.

**Figure 6:**
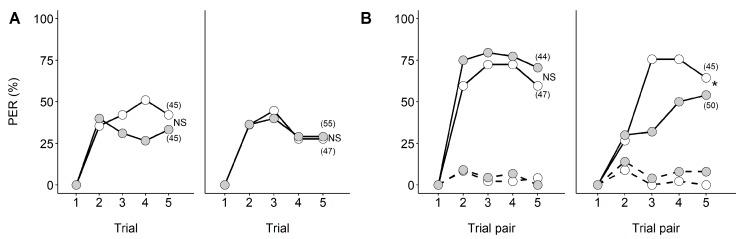
Effect of chronic exposure to GLY on young honey bee absolute and differential olfactory learning. Bees were reared in cages that offered sucrose solution alone (control, white circles) or with GLY (grey circles, 2.5 mg L^−1^) and were evaluated at five or nine days old (from left to right within each figure). (**A**) Percentage of bees that extended their proboscis (% PER) towards the conditioned stimulus (CS) in the training and testing phases of an absolute classical conditioning protocol. Minimal adequate models (GLMM with binomial structure): PER (training) ~ age + trial + cage + bee, PER (testing) ∼ TIU + cage. (**B**) Percentage of bees that extended their proboscis (% PER) towards the rewarded conditioned stimulus (CS+, solid lines) and the unrewarded conditioned stimulus (CS−, dashed lines) during the training and testing phases of a differential classical conditioning protocol. A discrimination index (DI) was defined based on PER towards the CS+ and the CS−. Minimal adequate models (GLMM with binomial structure): DI (training) ∼ GLY × age × trial + cage + bee, DI (testing) ∼ total individual uptake + age + cage. Numbers in brackets indicate sample size. NS: no significant differences. * indicates an effect of GLY (therefore, differences between its two levels), given that this factor is included in the minimal adequate model (MAM).

Olfactory associative learning is an ability present not only in foragers, but also in young in-hive workers. These pre-foraging bees receive olfactory and gustatory information regarding food sources through mouth to mouth contacts [58] and through stored food. Associations between these stimuli are established, and workers can retrieve these olfactory memories later on in life [57]. This skill can be tested through different conditioning procedures, each of which reveals cognitive processes of a different nature [45,46]. In an absolute olfactory conditioning, bees are trained to associate an odor with a sucrose reward. In a differential one, one odor is rewarded and another is presented alone. In both protocols PER towards an olfactory stimulus is recorded. Chronic exposure to GLY did not interfere with honeybee performance during an absolute olfactory conditioning (Figure 6A). Surprisingly, it affected the probability of PER towards the odors presented during a differential conditioning. This effect is age-dependent, as it was only evident in nine-day old workers. These honeybees showed an impoverished performance after being fed with GLY throughout their adult life (Figure 6B). The differential effect can be linked to honey bee age rather than exposure time because fourteen day old workers fed with GLY showed a similar performance to control bees [35].

It is interesting that GLY affects honeybee performance in a differential conditioning protocol, but not in a simple one. This indicates that the former is more appropriate for detecting subtle effects on behavior. This protocol not only puts honeybees to the test of associating an odor with a reward, but also of distinguishing it from another that is not coupled with reward. One hypothesis is that bees exposed chronically to GLY manifest difficulties in discriminating odors. Another is that the association between the stimulus and the reward is odor-unspecific. As the mechanism of action of GLY in honeybees is unknown, we cannot infer which structures or learning events are being affected. Yet, previous reports in caged honeybees exposed chronically to GLY reveal a decrease in antioxidants associated with carotenoid–retinoid system and in the enzymatic activity of acetylcholinesterase and phenoloxidase [65,66,67], which could explain, at least partly, the behavioral outputs observed. Impairment of olfactory cognitive skills due to herbicide exposure at a time in which honey bees are tuning their olfactory system in terms of rewarded and unrewarded associations could mean that these workers are less prepared as novice foragers. Altogether, honeybee colonies that are permanently exposed to this widely used herbicide are likely to show a deficit in information propagation and nectar distribution and, therefore, fall into disfavor.

## 5. Chronic Exposure of GLY and Its Effects on the Youngest Hive Mates: How Brood Development Is Affected by an Herbicide

Stored resources (honey and bee bread) are spread among all colony members including drones via trophallaxis [68,69] and used to feed brood in later larval stadia by nursing bees. Meanwhile, brood in early larval stadia and honey bee queens receive worker/royal jelly (mixture of different glandular secretions) by nursing bees [70]. Honey bees have four moults that allow their growth during the larval stage [71] before nursing bees seal their cells for pupation (120 h post-hatching) [72,73]. Each moult normally occurs approximately every 24 h. Adequate brood nutrition is essential to accomplish optimal development without long-term consequences in adults [74]. At that point, GLY could affect the fate of the colony depending on the exposure level and its impact on nutrition of reproductive castes and brood [16,75,76,77]. Thompson and co-workers (2014) show that larvae ingest GLY by detecting an average concentration of 11.9 ± 3.8 mg·kg^−1^ in larvae samples four days after spraying of the herbicide in surrounding vegetation [25].

**Figure 7:**
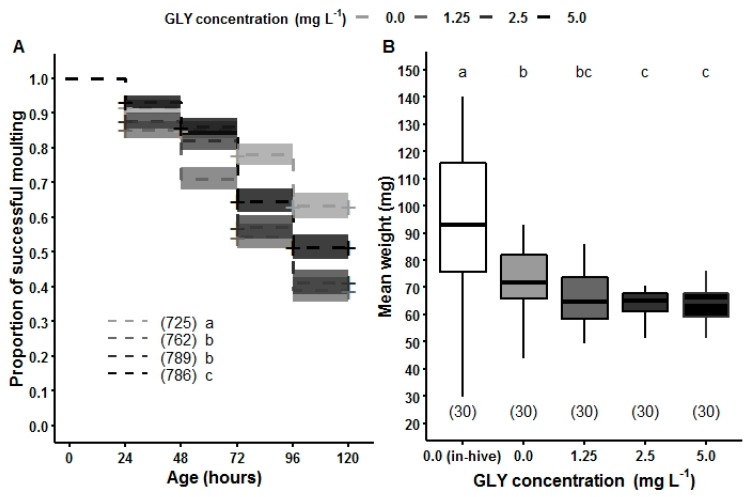
Effects of GLY on larval development of honey bee brood. Larvae reared in vitro were exposed to food contaminated with GLY (0 mg L^−1^, 1.25 mg L^−1^, 2.5 mg L^−1^, 5.0 mg L^−1^, greyscale) during the larval feeding period (0–120 h of age). (**A**) Proportion of larvae without delay in moulting and (**B**) larval weight at the end of the feeding period. White: data for control larvae reared in-hive. Curves of successful moulting are plotted with their confidence interval (95%) for each treatment. The + indicate time points with censored data. Main effects in development data: AFT model followed by a log-rank test for *post hoc* comparisons was carried out to analyse. Main effects in weight data: GLM followed by Tukey *post hoc* comparisons. Numbers in brackets indicate the number of larvae assessed for each treatment. Different letters indicate significant differences among treatments.

Detrimental effects in larval development, e.g., prolonged duration of early stadia, have been found in brood combs from hives close to crops with high levels of pesticides such as neonicotinoids, pyrethroids, and carbamates [78]. Indirect administration of GLY to brood via nursing by worker bees makes exposure conditions among larvae complex and heterogeneous. Therefore, Vazquez and co-workers [36] employed in vitro rearing to assess the effects of GLY on larval development. Even though this procedure does not account for social immunity, it allows homogenizing the exposure conditions and the herbicide dose. Brood provided with food containing GLY traces were more likely to present delayed moulting and weighed less than control brood (Figure 7).

The moulting process depends on growth rate, which is linked to feeding behavior. Thus, slight acidification of rearing food [36] and alteration of the gut microbiota [15,16,17] in adult bees due to GLY presence could have consequences in larvae. Triggering of stress compensatory mechanisms induces energy consumption [36,76], which could disrupt the moulting process due to a trade-off between growth and detoxification. Even if the in vitro procedure cannot be considered to completely reflect toxicity to larvae inside a hive due to differences in nutritional state and rearing context, it can help detect subtle adverse effects, as it was found. Nevertheless, this procedure does not mimic the pathway of exposure to agrochemicals under in-hive conditions, in which the frequency and amount of food offered vary according to larval demand and supply provided by nursing bees [79,80]. Consequently, honey bee response to GLY indicates that it is a stressor that affects larval development, but in vitro exposure acts as a worst-case scenario in which only the toxicity of the herbicide at the individual level can be assessed, with unknown long-term consequences at the colony level.

## 6. Conclusions and Perspectives

Nowadays, GMO crops engineered to resist herbicides are more available than conventionally bred resistant varieties [81]. In 2015, of the 179.9 million ha of global GMO crop area, about 84% contained crops that carried herbicide-resistant genes [82]. The dominant GMO crops are engineered for GLY tolerance [83]. However, it is worth mentioning that GLY is used also in many non-GMO crops situations and in many non-agronomical environments [6,13]. In this sense, 44% of the GLY used worldwide corresponds to non-agricultural use of GLY and includes even those countries that do not allow GM crops to be grown [6]. Since the mid-1990s, GLY use has increased exponentially in terms of volume of product applied, treated surface area and concentration used per surface unit [6]. In this panorama it is difficult to avoid linking the increased application of this herbicide in agriculture with the putative consequences of its use on biodiversity in general and on non-target organisms in particular.

Considering that a sentinel species gives information about the impact of subtle changes in the environment [84,85], by focusing on different aspects and moments of foraging related behaviors that take place during the honeybee life, it is possible to imagine the numerous risks to which an important pollinator is exposed to in agricultural environments where diverse technologies are implemented [6,13]. Honeybee foragers have been shown to have difficulties establishing and recalling elemental and non-elemental associations in a behavioral context that mimics a foraging flight situation (i.e., a conditioning procedure consisting of successive trials in which an odor and a reward are presented during training). The exposure to the herbicide might also impair learning and retrieval of memories necessary to perform successful homeward flights (evaluated by tracking the homeward flight of foragers using harmonic radar). Therefore, the ingestion of food containing high concentrations of GLY resulted in a higher proportion of disoriented foragers. Despite this, honeybees continued foraging from resources that contain GLY traces. These sublethal effects on their learning abilities could impact not only the foraging efficiency, but also the coordination of collective activities within the colony. The latter is well manifested in the behavioral abilities of young hive workers, since the presence of GLY in the circulating food could promote the loss of the sense of taste for sugar in nursing and food processing bees (shown by the testing of sucrose response thresholds on young workers) and finally affect food distribution within the hive. Constant inflow and storage of GLY contaminated nectar, which hive bees feed on, could even impair olfactory cognitive skills, which could mean that these workers are less prepared as novice foragers. Altogether, honeybee colonies that are permanently exposed to this widely used herbicide are likely to show a deficit in information propagation and nectar distribution. The last receiver in the food distribution chain is the brood, which could also receive food containing GLY in exposed colonies. This herbicide acts as a stressor that affects larval development (manifested in in-vitro exposure by a lower proportion of larvae achieving moulting success and reduced final weights), which could have implications for overall long-term colony survival.

Thus, the great versatility of the honey bee as an experimental model under controlled laboratory conditions allowed for the detection of different subtle effects on their sensory and cognitive abilities caused by the herbicide glyphosate that under natural conditions could be masked.
